# Cost-effectiveness analysis of non-invasive vagus nerve stimulation for the treatment of chronic cluster headache

**DOI:** 10.1186/s10194-016-0633-x

**Published:** 2016-04-22

**Authors:** James Morris, Andreas Straube, Hans-Christoph Diener, Fayyaz Ahmed, Nicholas Silver, Simon Walker, Eric Liebler, Charly Gaul

**Affiliations:** Cogentia Healthcare Consulting Ltd., Richmond House, 16-20 Regent Street, Cambridge, CB2 1DB UK; Ludwig Maximilian University of Munich, Marchioninistr 15, Munich, D81377 Germany; Department of Neurology and Headache Center, University Hospital Essen, Hufelandstrasse 55, 45122 Essen, Germany; Hull and Yorkshire Hospitals, Hull Royal Infirmary, Anlaby Road, Hull, HU3 2JZ UK; The Walton Centre for Neurology and Neurosurgery, Lower Lane, Liverpool, L9 7LJ UK; electroCore, LLC, 150 Allen Road, Suite 201, Basking Ridge, NJ 07920 USA; Migraine and Headache Clinic Königstein, Ölmühlweg 31, 61462 Königstein im Taunus, Germany

**Keywords:** Chronic cluster headache, Vagus nerve stimulation, Non-invasive, Cost-effectiveness, Germany, Pharmacoeconomics, United Kingdom

## Abstract

**Background:**

Cluster headache (CH) is a debilitating condition that is generally associated with substantial health care costs. Few therapies are approved for abortive or prophylactic treatment. Results from the prospective, randomised, open-label PREVA study suggested that adjunctive treatment with a novel non-invasive vagus nerve stimulation (nVNS) device led to decreased attack frequency and abortive medication use in patients with chronic CH (cCH). Herein, we evaluate whether nVNS is cost-effective compared with the current standard of care (SoC) for cCH.

**Methods:**

A pharmacoeconomic model from the German statutory health insurance perspective was developed to estimate the 1-year cost-effectiveness of nVNS + SoC (versus SoC alone) using data from PREVA. Short-term treatment response data were taken from the clinical trial; longer-term response was modelled under scenarios of response maintenance, constant rate of response loss, and diminishing rate of response loss. Health-related quality of life was estimated by modelling EQ-5D™ data from PREVA; benefits were defined as quality-adjusted life-years (QALY). Abortive medication use data from PREVA, along with costs for the nVNS device and abortive therapies (i.e. intranasal zolmitriptan, subcutaneous sumatriptan, and inhaled oxygen), were used to assess health care costs in the German setting.

**Results:**

The analysis resulted in mean expected yearly costs of €7096.69 for nVNS + SoC and €7511.35 for SoC alone and mean QALY of 0.607 for nVNS + SoC and 0.522 for SoC alone, suggesting that nVNS generates greater health benefits for lower overall cost. Abortive medication costs were 23 % lower with nVNS + SoC than with SoC alone. In the alternative scenarios (i.e. constant rate of response loss and diminishing rate of response loss), nVNS + SoC was more effective and cost saving than SoC alone.

**Conclusions:**

In all scenarios modelled from a German perspective, nVNS was cost-effective compared with current SoC, which suggests that adjunctive nVNS therapy provides economic benefits in the treatment of cCH. Notably, the current analysis included only costs associated with abortive treatments. Treatment with nVNS will likely promote further economic benefit when other potential sources of cost savings (e.g. reduced frequency of clinic visits) are considered.

**Trial registration:**

Clinicaltrials.gov identifier NCT01701245, 03OCT2012.

## Background

Cluster headache (CH) is a debilitating condition associated with intense pain and cranial autonomic symptoms, which cause marked disability [1]. The disorder adversely affects quality of life [2] and is associated with substantial health care costs (more than €11,000 per year) [3]. The condition can be chronic or episodic. Both direct costs (e.g. medication, clinic visits) and indirect costs (e.g. reduced work capacity) have been found to be substantially higher for patients with chronic CH (cCH) than for those with episodic CH [3]. Few drugs (e.g. subcutaneous [SC] sumatriptan, intranasal [IN] zolmitriptan, and dihydroergotamine [DHE] injection) are approved by various regulatory agencies for abortive treatment [4, 5]. Lithium is approved for CH prophylaxis in Germany [6] and is used off-label in other areas. Other agents such as verapamil and topiramate are also used off-label despite a lack of rigorous, well-controlled studies to support their use in the prevention of CH attacks [7–9]. Although short-term methylprednisone therapy may be effective in CH prophylaxis, several safety concerns preclude its long-term use [8].

Vagus nerve stimulation (VNS) is a neuromodulatory technique that is well established for epilepsy and depression and has been applied to a variety of other disorders including Alzheimer disease, migraine, and CH [10–12]. It is thought to suppress pain through inhibition of vagal afferents in the trigeminal nucleus caudalis (TNC) [13] and by blocking or reversing increases in TNC glutamate levels [14]; VNS has also been implicated in modulation of the cholinergic anti-inflammatory pathway [15–17].

In an initial open-label study (*N* = 19), non-invasive vagus nerve stimulation (nVNS) was found to be effective in the prevention and treatment of CH [11]. Subsequently, a larger (*N* = 97), prospective, open-label, randomised study (PREVA [18]) evaluated the safety and efficacy of adjunctive treatment with a novel nVNS device (gammaCore^®^) in patients with cCH. In the PREVA trial, compared with standard of care (SoC) alone, adjunctive nVNS (nVNS + SoC) was associated with significantly greater decreases from baseline in the number of CH attacks per week and the use of abortive medications. Compared with SoC alone, nVNS + SoC was also associated with a significantly higher response rate (i.e. the proportion of participants with a ≥50 % reduction from baseline in the number of CH attacks per week; 40 % for nVNS + SoC vs 8.3 % for SoC alone, *P* < 0.001) and significantly greater improvements from baseline in quality-of-life measures, with no serious treatment-related adverse events.

The present analysis was undertaken to quantify the economic impact of nVNS therapy in patients with cCH. By developing a pharmacoeconomic model and applying it to data from the PREVA study, we evaluated whether nVNS is a cost-effective treatment option compared with the current standard practice in a European setting. Analysis using German costs is the focus of this paper because Germans represented the largest proportion of PREVA participants. To corroborate our findings and widen their applicability, we conducted a similar analysis using UK costs, which is briefly described in the Discussion section.

## Methods

### Study design

**Fig. 1:**
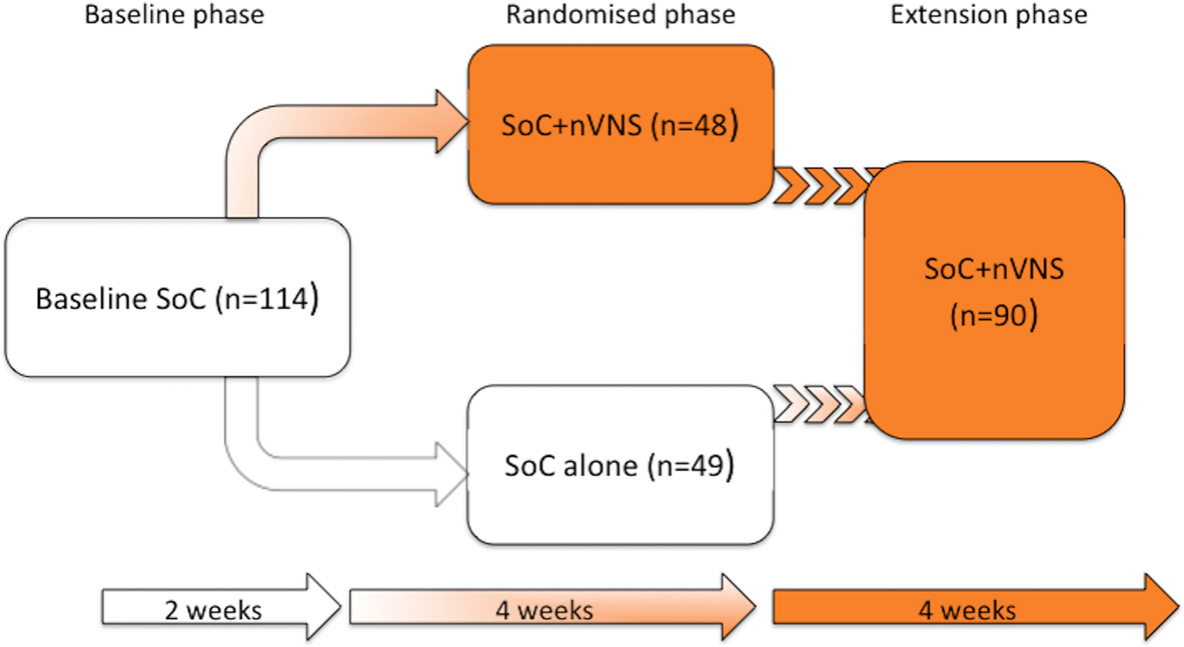
PREVA study [18] design. Abbreviations: *nVNS* non-invasive vagus nerve stimulation, *SoC* standard of care

The principal data source for this analysis was the PREVA study (clinicaltrials.gov identifier NCT01701245), which compared the effectiveness of nVNS added to SoC with that of SoC alone as prophylactic therapy for cCH. For each participant, SoC was individualised and typically included prophylactic medications (e.g. verapamil, lithium) and abortive agents (e.g. inhaled oxygen, triptans). The study design (Fig. 1) and methodology of PREVA have been described in detail previously [18]. The PREVA study was conducted in accordance with the principles and requirements of the Declaration of Helsinki, Good Clinical Practices, and clinical trial registration. All PREVA investigators obtained institutional review board approval, and all PREVA participants provided written informed consent.

### Model structure and parameter estimates

**Fig. 2:**
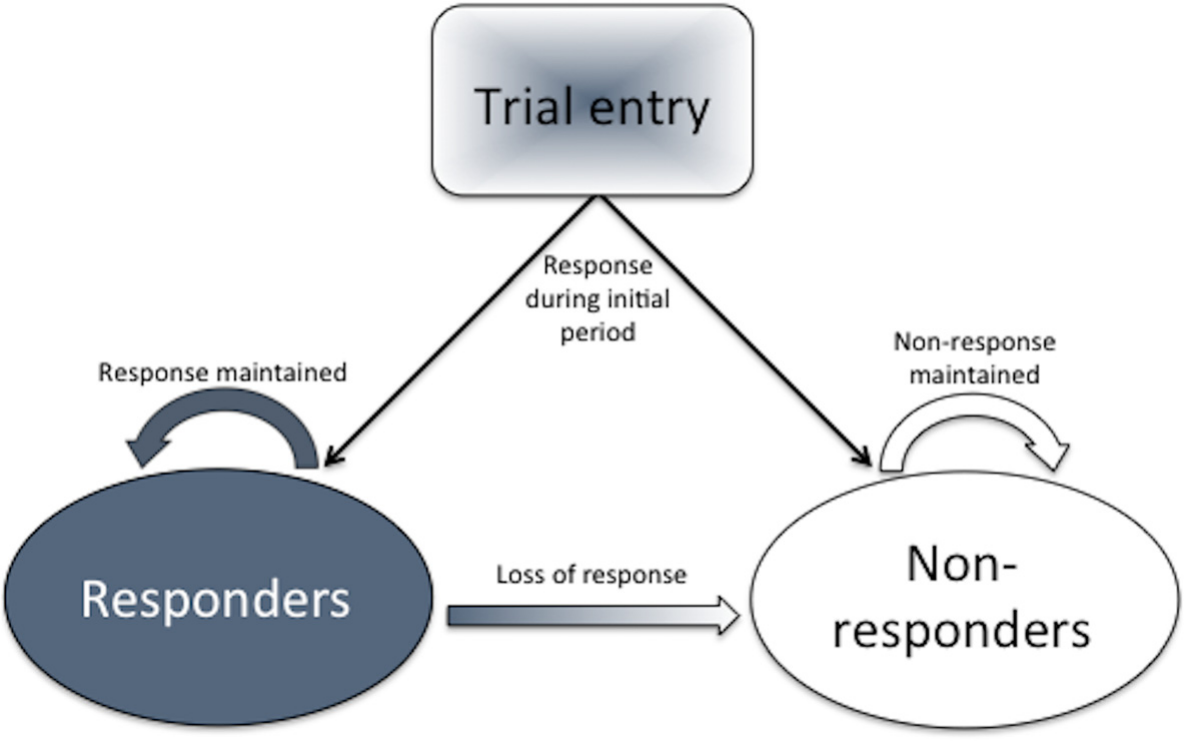
Pharmacoeconomic model structure. *Response* was defined as a ≥50 % reduction from baseline in the number of CH attacks during the randomised period (or during month 2 in the case of 4 late responders). Responders in the SoC group were modelled as non-responders beyond the randomised phase. Probability of response was modelled for the base case (response maintained) and for the following alternative scenarios: 1) constant rate of response loss, 2) diminishing rate of response loss, and 3) no initial response in the SoC group. Abbreviations: *CH* cluster headache; *SoC* standard of care

Figure 2 depicts the 1-year model that was used to estimate the cost-effectiveness of adjunctive nVNS therapy from the German statutory health insurance perspective*.* Model parameter estimates were derived from data on the efficacy of nVNS and the use of abortive medications from the randomised phase of PREVA. *Treatment response* was defined as ≥50 % reduction from baseline in the number of CH attacks per week. Beyond the randomised phase, responders in the SoC group were assumed to be non-responders, and non-responders in the nVNS + SoC group were assumed to discontinue prophylactic treatment with nVNS but continue use of abortive treatments. Four late responders in the nVNS + SoC group (i.e. patients who were not classified as responders during the randomised phase but responded during the extension phase) were included as responders in the base case. An alternative scenario in which the 4 late responders were classified as non-responders was also modelled in a sensitivity analysis.

To estimate the probability of response in the base case analysis, subjects from the nVNS + SoC group who were responders throughout the extension phase were assumed to maintain this response until the end of the model time horizon (1 year). In addition to the base case analysis, 3 alternative scenarios were explored. An exponential survival curve function was fitted to data from patients in the nVNS + SoC group on the basis of their response statuses at the end of the randomised phase and at the end of the extension phase. In the first alternative scenario, the exponential function was used to predict patient response status beyond 4 weeks (i.e. beyond the randomised phase) assuming a constant monthly rate (~31 %) of response loss throughout the course of the model. The second scenario was modelled assuming a diminishing rate of response loss; that is, the rate at which response was lost beyond 4 weeks (as predicted by the exponential function) was reduced by a fixed percentage (10 %) each month. In the final scenario, no patients in the SoC-alone group were assumed to have responded initially, and all other assumptions were the same as in the base case.

Benefits in this analysis were defined as quality-adjusted life-years (QALY). Health-related quality of life (HRQoL) for responders and non-responders was estimated by modelling EQ-5D™ index data from PREVA in an ordinary least squares regression analysis to control for potential imbalances at baseline between treatment arms. Results from the regression analysis suggested that response was associated with an increase of 0.2366 in EQ-5D index score and that nVNS therapy (regardless of response) was associated with an increase of 0.01246 in EQ-5D index score. Using the German tariff, HRQoL utility scores were estimated for responders and non-responders and applied to the model states (the UK tariff was applied for the UK analysis).

Data on abortive medication use from the last 14 days of the PREVA randomised phase (Table 1) were used to assess health care resource utilisation. Patients in the nVNS + SoC group who maintained responder status were assumed to continue using the same amount of resources as those observed in the overall nVNS + SoC group during the randomised phase. Non-responders were assumed to have the same resource use as that observed in the SoC group during the randomised phase. Unit costs for nVNS, triptans, and inhaled oxygen are shown in Table 2. The nVNS use cost was the listed price in Germany, and unit costs for IN zolmitriptan and SC sumatriptan were determined from the Lauer-Taxe^®^ [19]. Costs for inhaled oxygen were derived using the estimated daily cost for oxygen from a previous study [3] and data from the baseline phase of PREVA.

**Table 1:** Abortive medication use during the last 14 days of the PREVA randomised phase

Abortive medication	No. of uses, mean (SD)	
	nVNS + SoC (*n* = 45)	SoC alone (*n* = 48)
IN zolmitriptan	1.6 (5.5)	1.3 (3.6)
SC sumatriptan	2.8 (4.0)	7.5 (9.6)
Inhaled oxygen	6.5 (11.1)	10.8 (15.3)

Abbreviations: *IN* intranasal, *nVNS* non-invasive vagus nerve stimulation, *SC* subcutaneous, *SD* standard deviation, *SoC* standard of care

**Table 2:** Unit cost of treatments

Treatment	Description	Cost per dose, €
IN zolmitriptan	AscoTop^**®**^ Nasal 5 mg/Dosis Nasenspray, Solution €86.22: 6 single-dose nasal sprays, PZN 03107201	14.07^a^
SC sumatriptan	Sumatriptan-Hormosan Inject 6 mg/0.5-mL Solution €64.40: 2 pre-filled syringes, PZN 04700154	31.31^a^
Inhaled oxygen	Estimated cost per CH attack	2.87
nVNS	gammaCore device pre-loaded with 300 stimulations	0.87

Abbreviations: *IN* intranasal, *nVNS* non-invasive vagus nerve stimulation, *SC* subcutaneous

^a^Prices include mandatory pharmacy discount of €1.77 per pack

Published prices for zolmitriptan and sumatriptan were taken from Lauer-Taxe (cheapest available price selected) [19]. Price for oxygen was estimated using daily cost from Gaul et al [3]

All economic models are associated with uncertainty; we used a conventional method to reflect this in the analysis by developing a probabilistic model using a Markov chain Monte Carlo simulation to quantify how parameter uncertainty affects model results (i.e. the cost-effectiveness estimates for nVNS + SoC) [20, 21] (Table 3). Distributions for each model parameter of interest were estimated in line with best practice. A probabilistic analysis with 1000 simulations for each scenario was conducted, and mean values from this analysis were calculated. Each simulation was plotted on the cost-effectiveness plane to show the spread of results.

**Table 3:** Parameters for the probabilistic sensitivity analysis

Parameter	Mean	SE	Distribution
Probability of response with nVNS + SoC	0.489	0.074	Beta
Probability of response with SoC alone	0.083	0.039	Beta
Probability of discontinued response	0.310	0.378	Normal^a^
Utility score (nVNS + SoC responder)	0.772	NA	Multivariate normal
Utility score (nVNS + SoC non-responder)	0.536	NA	Multivariate normal
Utility score (SoC alone responder)	0.760	NA	Multivariate normal
Utility score (SoC alone non-responder)	0.523	NA	Multivariate normal
*Resource use per 14 days*			
With nVNS + SoC			
Zolmitriptan	1.6	0.82	Gamma
Sumatriptan	2.8	0.60	Gamma
Oxygen	6.5	1.65	Gamma
With SoC alone			
Zolmitriptan	1.3	0.52	Gamma
Sumatriptan	7.5	1.38	Gamma
Oxygen	10.8	2.21	Gamma

Abbreviations: *NA* not applicable, *nVNS* non-invasive vagus nerve stimulation, *SE* standard error, *SoC* standard of care

^a^Based on exponential survival function

## Results

### Base case

**Fig. 3:**
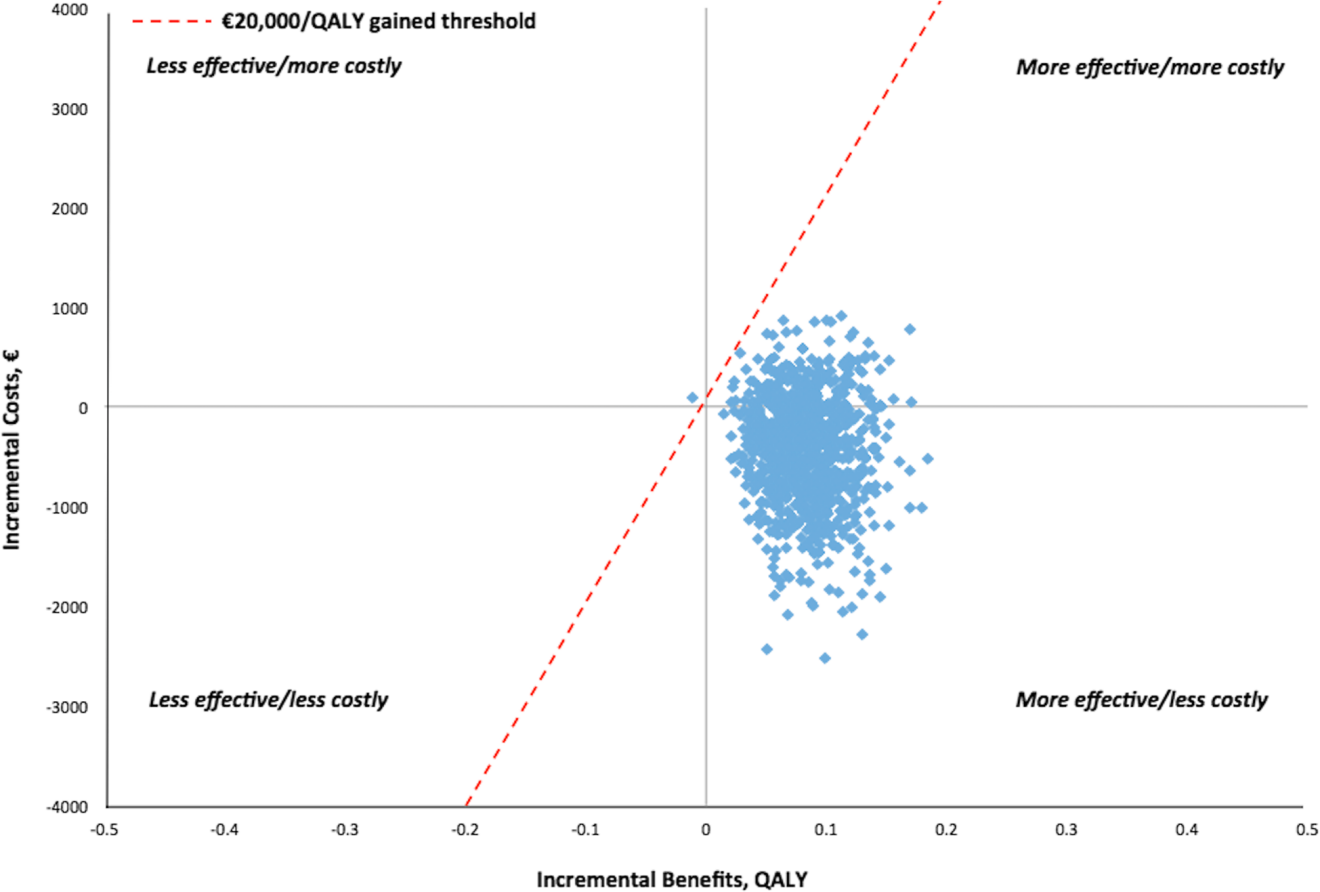
Plot of the base case model simulations (cost-effectiveness plane). Abbreviation: *QALY* quality-adjusted life-year

**Fig. 4:**
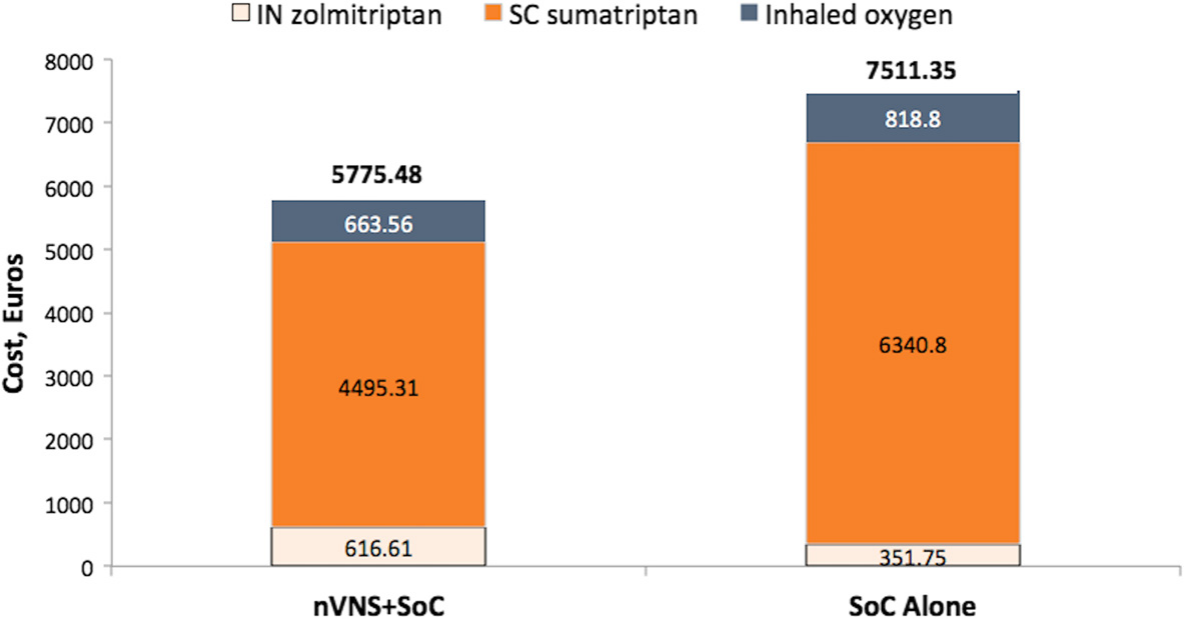
Breakdown of modelled 1-year costs of abortive medications by category. Abbreviations: *IN* intranasal; *nVNS* non-invasive vagus nerve stimulation; *SC* subcutaneous; *SoC* standard of care

For the German base case, the analysis resulted in mean expected costs of €7096.69 for nVNS + SoC and €7511.35 for SoC alone and mean QALY of 0.607 for nVNS + SoC and 0.522 for SoC alone. Thus, nVNS + SoC appears to generate greater health benefits for lower overall cost (Table 4). Approximately 80 % of the probabilistic simulations resulted in cost savings for nVNS + SoC (versus SoC alone), and the vast majority of the simulations plotted fell below the commonly used €20,000/QALY gained threshold (i.e. the amount that commissioners of health care services are willing to pay per additional unit of health with new technologies) (Fig. 3) [22–24]. Overall abortive medication costs were 23 % lower in the nVNS + SoC group than in the SoC-alone group (Fig. 4). Compared with the SoC-alone group, the nVNS + SoC group had 29 % lower SC sumatriptan costs, 19 % lower inhaled oxygen costs, and 75 % higher IN zolmitriptan costs.

**Table 4:** Base case^a^ cost-effectiveness analysis

Treatment group	Mean cost, €	Mean QALY	ICER^b^
nVNS + SoC	7096.96	0.607	nVNS dominant over SoC^c^
SoC alone	7511.35	0.522	

Abbreviations: *ICER* incremental cost-effectiveness ratio, *nVNS* non-invasive vagus nerve stimulation, *QALY* quality-adjusted life-year, *SoC* standard of care

Probabilistic estimates are based on mean results across all Monte Carlo simulations [21]

^a^In the base case, subjects in the nVNS + SoC group who responded through the extension phase were assumed to maintain response

^b^The expense of gaining an additional QALY with adjunctive nVNS therapy (vs SoC alone)

^c^Indicates that adjunctive nVNS therapy was more effective and cost saving than SoC alone

### Alternative scenarios and sensitivity analysis

Altering the model by varying the likelihood for loss of response in either group had little effect on the relative cost-effectiveness of nVNS (Table 5). In the alternative scenarios explored, the percentages of the probabilistic simulations that resulted in cost savings for nVNS + SoC (versus SoC alone) were ~71 % for *constant rate of response loss* and ~79 % for both *diminishing rate of response loss* and *no response for SoC*. Results from the sensitivity analysis suggest that exclusion of the 4 late responders to nVNS (i.e. designating them as non-responders in the model) had a modest impact on cost-effectiveness. For all scenarios modelled in the sensitivity analysis, nVNS + SoC was more effective and cost saving (Table 6).

**Table 5:** Cost-effectiveness analysis for alternative scenarios

Scenario			
Treatment group	Mean cost, €	Mean QALY	ICER^a^
Constant rate of response loss			
nVNS + SoC	7377.41	0.558	nVNS dominant over SoC^b^
SoC alone	7518.56	0.526	
Diminishing rate of response loss			
nVNS + SoC	7141.30	0.599	nVNS dominant over SoC^b^
SoC alone	7508.98	0.525	
No response for SoC			
nVNS + SoC	7085.34	0.610	nVNS dominant over SoC^b^
SoC alone	7507.94	0.524	

Abbreviations: *ICER* incremental cost-effectiveness ratio, *nVNS* non-invasive vagus nerve stimulation, *QALY* quality-adjusted life-year, *SoC* standard of care

Probabilistic estimates are based on mean results across all Monte Carlo simulations [21]

^a^The expense of gaining an additional QALY with adjunctive nVNS therapy (vs SoC alone)

^b^Indicates that adjunctive nVNS therapy was more effective and cost saving than SoC alone

**Table 6:** Cost-effectiveness sensitivity analysis (4 late responders excluded)

Scenario			
Treatment group	Mean cost, €	Mean QALY	ICER^a^
Response maintained			
nVNS + SoC	7380.93	0.566	nVNS dominant over SoC^b^
SoC alone	7540.28	0.536	
Constant rate of response loss			
nVNS + SoC	7392.09	0.550	nVNS dominant over SoC^b^
SoC alone	7440.13	0.539	
Diminishing rate of response loss			
nVNS + SoC	7279.89	0.560	nVNS dominant over SoC^b^
SoC alone	7385.29	0.537	

Abbreviations: *ICER* incremental cost-effectiveness ratio, *nVNS* non-invasive vagus nerve stimulation, *QALY* quality-adjusted life-year, *SoC* standard of care

^a^The expense of gaining an additional QALY with adjunctive nVNS therapy (vs SoC alone)

^b^Indicates that adjunctive nVNS therapy was more effective and cost saving than SoC alone

## Discussion

The treatment of CH is challenging, and many of the commonly used abortive and preventive medications are associated with serious safety risks, poor tolerability, and/or marginal efficacy. For acute treatment, triptans are contraindicated in patients with cardiovascular disease [25, 26]. Drug costs or restrictions on prescribing and/or coverage may further limit triptan accessibility for many patients [27, 28]. Long-term frequent use of triptans, as may be needed for cCH management, can in turn lead to the development of medication overuse headache [29, 30], which, although rare, has been reported in patients with CH [31, 32]. Oxygen may delay rather than abort CH attacks in some patients and has portability limitations [25, 26], and DHE may be associated with fibrosis (e.g. cardiac, pulmonary, pleural), ergotism, and chest tightness [26, 33]. For prophylactic treatment, verapamil has a high potential for drug interactions, and the large dosages required for CH treatment are associated with adverse cardiac events such as arrhythmias, as well as oedema [26]. Lithium requires progressive titration and frequent drug-level monitoring because of its narrow therapeutic window and the risk of toxicity [25, 26, 34], and topiramate is often poorly tolerated owing to its cognitive side effects [26]. Thus, more practical and cost-effective treatment approaches for CH are needed. Results from the PREVA study [18] suggest that in addition to reducing the frequency of CH attacks, adjunctive nVNS therapy may decrease the need for abortive medications and improve quality of life in patients with cCH. The current pharmacoeconomic analysis indicates that adjunctive nVNS is likely to result in cost savings when compared with SoC alone. Notably, the present analysis was conservative in that it included only the costs associated with use of abortive medications without accounting for other potential sources of cost savings (e.g. reduced frequency of clinic visits, fewer hospitalisations, increased productivity).

Currently, there are few good options for acute or prophylactic treatment of CH. Neuromodulation methods such as sphenopalatine ganglion (SPG) stimulation and occipital nerve stimulation (ONS) have shown some promise in CH prevention, but most studies of these techniques have been small and/or have lacked control arms [35, 36]. Furthermore, SPG and ONS are invasive, expensive, and associated with risks inherent with implanted devices (e.g. infection, pain at the site of implantation, electrode migration). The findings that nVNS is effective in cCH prophylaxis [18], is not associated with risks that are inherent in invasive neuromodulation methods, and offers cost savings over the current standard practice suggest that this therapy warrants a prominent place in the management of cCH.

The current analysis is subject to certain limitations. The PREVA study provided data from an 8-week period, which were extrapolated to assess cost-effectiveness over 1 year. Although there have been few cost-effectiveness evaluations of neuromodulatory techniques for the treatment of primary headache disorders, such studies have generally included time horizons of at least 3 years [37–39]. Considering the time frame of PREVA, a 1-year time horizon was chosen for this analysis to preserve robustness and to avoid introducing unnecessary uncertainty. As in patients with epilepsy [40], evidence suggests that patients with headache may have improved response to VNS with longer-term treatment [41, 42]. Although increases in response rate with long-term VNS have yet to be explored in CH, the current analysis could be viewed as conservative because the duration of PREVA may not have allowed demonstration of the full benefit of nVNS.

Recently, the National Institute for Health and Care Excellence (NICE) Interventional Procedures Advisory Committee noted that the relapsing/remitting nature of CH and migraines as well as the potential for placebo effects should be considered when interpreting evidence of treatment efficacy for these conditions [43]. Indeed, because periods of relapse and remission are common among patients with primary headache disorders, research in this area may be susceptible to regression artefacts [44, 45]. However, the PREVA study included data from patients with cCH only. By *International Classification of Headache Disorders* definition [46], cCH is not associated with extended periods of remission (i.e. ≥1 month), suggesting that the phenomenon of regression to the mean (e.g. aberrantly high attack frequency at baseline followed by a decrease in attack frequency regardless of treatment group) would not be expected. Because the PREVA study lacked a sham treatment group, the degree to which the placebo effect might have contributed to the cost-effectiveness of nVNS is unclear. Nevertheless, the clinically relevant design of the PREVA study was valuable in that it allowed for observation of medication use in a control group that likely reflects real-world use.

As with any probabilistic analysis, some degree of uncertainty is inherent in the current investigation. To address this, a sensitivity analysis and a range of alternative scenarios were included, and results from all of these suggested that nVNS + SoC was more effective and cost saving than SoC alone. Results were relatively insensitive to assumptions about late responders in the nVNS + SoC arm. In the sensitivity analysis, where the 4 late-responding patients were classified as non-responders, nVNS + SoC was dominant over SoC alone in all modelled scenarios.

The current analysis cannot be directly extrapolated across all of Europe because it evaluates cost-effectiveness from a German health insurance perspective. To explore the generalisability of our findings, we conducted the same analysis from a UK perspective and found similar results. For the base case, the probabilistic analysis resulted in mean expected costs of £5409.83 for nVNS + SoC and £5393.31 for SoC alone and mean QALY of 0.538 for nVNS + SoC and 0.438 for SoC alone. The incremental cost-effectiveness ratio of nVNS + SoC was £166.12, and 47 % of the probabilistic simulations resulted in cost savings for nVNS + SoC over SoC alone (J. Morris, unpublished data, 2016). The degree to which these results can be generalised to other countries may vary depending on specific drug prices and the availability of generic medications in those markets.

Lastly, the current cost-effectiveness projections included only the costs associated with the use of abortive treatments. This suggests that our analysis is conservative, as data on additional health care resource use (e.g. clinic visits) would likely lead to a disproportionate cost increase for the SoC-alone group. Likewise, potential health benefits from decreased use of abortive medications (e.g. drug-related side effects) and effects on indirect costs (e.g. increased work capacity), which could further enhance the economic profile of nVNS, were not considered herein. The economic benefits of nVNS could be established with greater certainty by incorporating additional cost components into future studies.

## Conclusions

The current study provides evidence of the efficacy and economic benefits of nVNS therapy for patients with cCH in the context of the German and UK health care systems. In all scenarios modelled, nVNS was more cost-effective than the current standard practice. These findings are especially meaningful given the substantial economic burden associated with CH [3] and considering that new technologies are cited as major drivers of increasing health care expenditures [47, 48]. Our results suggest that new technologies such as nVNS may help decrease overall treatment costs, information that likely will be important to clinicians, patients, and payers when treatment decisions are made.

### Availability of data and materials

Clinical data from the PREVA study are available in the following publication: Gaul C, et al (2015) Non-invasive vagus nerve stimulation for PREVention and Acute treatment of chronic cluster headache (PREVA): a randomised controlled study [Published online September 21]. Cephalalgia. doi:10.1177/0333102415607070.

Economic data supporting the conclusions in this manuscript are on file at Cogentia Healthcare Consulting Ltd. and electroCore, LLC, and are confidential in order to support economic filings in the affected countries.

## Abbreviations

cCH: chronic cluster headache
CH: cluster headache
DHE: dihydroergotamine
HRQoL: health-related quality of life
IN: intranasal
NICE: National Institute for Health and Care Excellence
nVNS: non-invasive vagus nerve stimulation
ONS: occipital nerve stimulation
QALY: quality-adjusted life-year
SC: subcutaneous
SoC: standard of care
SPG: sphenopalatine ganglion
TNC: trigeminal nucleus caudalis
VNS: vagus nerve stimulation
